# Trends, Seasonality, and the Impact of COVID-19 on Clinical *Staphylococcus aureus* and MRSA Isolates in Western Mexico (2016–2025): A Time-Series Analysis at a University Referral Hospital

**DOI:** 10.3390/antibiotics15030242

**Published:** 2026-02-25

**Authors:** Jaime Briseno-Ramírez, Pedro Martínez-Ayala, Adolfo Gómez-Quiroz, Brenda Berenice Avila-Cardenas, Brian Rafael Rubio-Mora, Roberto Miguel Damian-Negrete, Ana María López-Yáñez, Leonardo García-Miranda, Carlos Roberto Álvarez-Alba, Judith Carolina De Arcos-Jiménez

**Affiliations:** 1 Hospital Civil de Oriente, Tónala 45425, Mexico; jaime.briseno@academicos.udg.mx (J.B.-R.); ana.lopez@academicos.udg.mx (A.M.L.-Y.); 2 División Salud, Centro Universitario de Tlajomulco, Universidad de Guadalajara, Tlajomulco de Zuñiga 45641, Mexico; roberto.damian@cutlajomulco.udg.mx (R.M.D.-N.); leonardo.garcia8005@alumnos.udg.mx (L.G.-M.); 3 Antiguo Hospital Civil de Guadalajara “Fray Antonio Alcalde”, Guadalajara 44280, Mexico; pedro.martinez@cucs.udg.mx (P.M.-A.); agomezq@hcg.gob.mx (A.G.-Q.); 2019122@mail.hcg.udg.mx (B.B.A.-C.); brubio@hcg.gob.mx (B.R.R.-M.); carlosralvareza@gmail.com (C.R.Á.-A.)

**Keywords:** *Staphylococcus aureus*, methicillin-resistant *Staphylococcus aureus*, antimicrobial resistance, time series analysis, interrupted time series, COVID-19 pandemic

## Abstract

Background/Objectives: Methicillin-resistant *Staphylococcus aureus* (MRSA) remains a major cause of both community-onset and hospital-acquired infections, yet longitudinal data from Latin American hospitals spanning the COVID-19 pandemic are scarce. We characterized temporal trends, seasonality, and the impact of the COVID-19 pandemic on MRSA prevalence and incidence density among clinical *S. aureus* isolates at a tertiary-care hospital in western Mexico over 9.5 years. Methods: We analyzed 6625 non-duplicate clinical *S. aureus* isolates (6609 with valid resistance data) from June 2016 to December 2025. Temporal trends were assessed using Mann–Kendall tests, Theil–Sen estimation, and binomial generalized linear models. Seasonality was evaluated through STL decomposition, generalized additive models, and Fourier analysis. An interrupted time series (ITS) model with GLS-AR(1) and Newey–West corrections compared three COVID-19 phases: pre-pandemic (2016–2020), high viral circulation (2020–2022), and post-peak stabilization (2022–2025). Exposure-adjusted incidence densities (per 1000 patient-days) were analyzed in parallel. Results: MRSA prevalence declined from 28.1% pre-pandemic to 14.0% post-peak (Mann–Kendall *z* = −9.03, *p* < 0.001; OR = 0.85 per year, 95% CI: 0.829–0.871). MRSA incidence density decreased by 50%, from 1.27 to 0.63 per 1000 patient-days, while aggregate *S. aureus* incidence density remained stable (*z* = −0.17, *p* = 0.868). The ITS joint Wald test confirmed a significant cumulative shift in MRSA trajectory post-pandemic (*p* = 0.019 counts; *p* = 0.012 incidence density), with a significant post-peak level drop (*p* = 0.008). *S. aureus* exhibited moderate seasonality peaking in May–July (GAM edf = 7.26, *p* < 0.001), whereas MRSA showed only marginal seasonal variation. Conclusions: MRSA declined markedly across the study period, with the steepest reduction following the Omicron peak. The decline persisted after adjustment for pandemic-related fluctuations in hospital volume, supporting periodic reassessment of empiric anti-MRSA prescribing policies in similar settings.

## 1. Introduction

*Staphylococcus aureus* is a globally prevalent pathogen that contributes significantly to the burden of disease across diverse populations and a wide range of clinical settings [1]. In acute-care hospitals, *S. aureus* encompass both community-onset infections diagnosed at or shortly after admission and hospital-acquired infections arising during hospitalization, reflecting the pathogen’s capacity to cause disease across the full spectrum of healthcare exposure [2,3]. Population-based surveillance in the United States has shown that community-onset cases (including healthcare-associated, community-onset and community-associated infections) account for approximately 80% of all invasive MRSA episodes identified among hospitalized patients, with hospital-onset infections representing the remaining 20% [4,5]. *S. aureus* infections are associated with significant excess mortality (20.2% at one year), increased risk of long-term disability, prolonged hospital stays (by an average of 12 days), and increased healthcare costs [6,7,8].

The incidence of MRSA infections has declined markedly in high-resource settings over the past two decades, driven primarily by reductions in hospital-onset cases. In U.S. Department of Veterans Affairs medical centers, overall *S. aureus* infections fell by 43% between 2005 and 2017, with MRSA declining by 55% and methicillin-susceptible *S. aureus* (MSSA) by 12% [7]. Although the initial decline was substantial, more recent surveillance indicates that the downward trend in hospital-onset MRSA has plateaued, while community-onset MRSA–which had been increasing before the pandemic–showed a temporary decrease during 2020–2022, likely related to COVID-19 mitigation measures [2,4,5,8]. The traditional distinction between community-associated (CA-MRSA) and healthcare-associated (HA-MRSA) lineages has become increasingly blurred, as genotypically community-associated clones such as USA300 now cause nosocomial infections, and HA-MRSA clones circulate in community settings [9,10].

In Latin American hospitals, recent multicenter studies indicate that MRSA continues to represent a substantial proportion of *S. aureus* infections, accounting for approximately 44–45% of *S. aureus* bloodstream infections across multiple countries [11]. However, marked heterogeneity exists in MRSA prevalence by country and region, with some areas reporting higher rates (e.g., Peru) and others lower (e.g., Venezuela) [12]. The molecular epidemiology of MRSA in the region is also evolving: community-associated clones, particularly the USA300 Latin American variant (USA300-LV, ST8-SCCmec IVc), have emerged alongside traditional healthcare-associated lineages such as the Chilean/Cordobes clone (ST5-SCCmec I), and in some countries have begun to displace hospital-acquired strains [11,12,13]. In Argentina, national surveillance data indicate that while the overall incidence of *S. aureus* infections has increased—driven primarily by MSSA—the incidence of MRSA infections has remained relatively stable in recent years [14]. MRSA prevalence in Mexican hospitals has remained substantial, with recent multicenter surveillance reporting methicillin resistance rates in *S. aureus* as high as 21–27% in clinical isolates from diverse regions and hospital types [15,16], and recent genomic characterization identifying CC5 and CC8 as the dominant clonal complexes [17]. Comparative studies of resistance patterns in trauma patients treated in Mexican hospitals versus those in the United States further highlight the higher frequency of MRSA and other multidrug-resistant organisms in Mexican healthcare settings, emphasizing the need for robust infection control and antimicrobial stewardship [18]. Despite these reports, most available data from the region are derived from cross-sectional or short-term multicenter surveys; single-center studies providing the longitudinal resolution needed to characterize temporal dynamics across the pre-pandemic, pandemic, and post-pandemic periods remain scarce.

Prior to the COVID-19 pandemic, multiple studies documented a gradual decline in the incidence and prevalence of MRSA in hospital settings, an effect attributed to sustained infection-control practices and antimicrobial stewardship efforts [5,19]. During the pandemic period, MRSA trends proved heterogeneous across epidemiologic categories and geographic settings [20,21,22]. In the United States, hospital-onset MRSA bacteremia increased by approximately 40% in 2021 relative to prepandemic levels–an increase largely attributable to patients with recent COVID-19–whereas community-associated MRSA declined, possibly reflecting pandemic mitigation measures [5]. Several investigations have reported reductions in MRSA acquisition ranging from 12.7% to 41% following the implementation of enhanced infection control measures, including intensified hand hygiene and expanded use of personal protective equipment [23,24,25,26,27,28,29]. In contrast, a large multicenter cohort study observed a 31.5% increase in hospital-onset MRSA and other antimicrobial-resistant (AMR) infections during the peak of the COVID-19 pandemic (March 2020 to February 2022) compared to the pre-pandemic period [30]. Although overall AMR rates returned to baseline as the pandemic subsided, hospital-onset AMR infections—including MRSA—remained elevated above pre-pandemic levels [30]. Similar increases have been reported by other authors [20,31].

Because longitudinal data on *S. aureus* resistance trends from Latin American hospitals spanning the COVID-19 pandemic are scarce, this study aims to characterize the long-term epidemiology of clinical *S. aureus* (including MRSA) isolates–encompassing both community-onset and hospital-acquired infections–at a tertiary-care referral university hospital in western Mexico over a 9.5-year period, to identify and model underlying seasonal patterns, and to assess the pandemic’s impact on these trends, with the goal of informing future infection-prevention and antimicrobial-stewardship strategies.

## 2. Results

### 2.1. Demographic, Clinical, and Microbiological Characteristics of the Study Population

**Figure 1:**
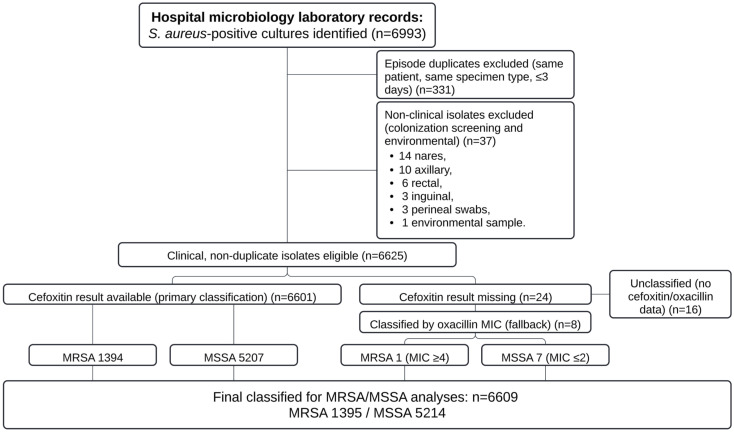
Study flow diagram for *Staphylococcus aureus* isolate selection and MRSA/MSSA classification.

From an initial dataset comprising 6993 *Staphylococcus aureus*–positive cultures, 331 duplicate episodes were excluded (same patient, same specimen type, and within ≤3 days), and 37 non-clinical isolates were removed (colonization screening and environmental samples). This yielded 6625 eligible clinical, non-duplicate isolates collected between 30 June 2016 and 31 December 2025. Methicillin resistance was determined primarily by cefoxitin screening, which was available for 6601 isolates. For 24 isolates with missing cefoxitin results, oxacillin minimum inhibitory concentration (MIC) data were used as a predefined fallback, allowing classification of 8 additional isolates. The remaining 16 isolates lacked both cefoxitin and oxacillin data and were excluded from MRSA/MSSA subgroup analyses but were retained for overall *S. aureus* burden estimates. Therefore, 6609 isolates (99.8% of eligible clinical isolates; 94.5% of the initial dataset) had a valid methicillin-resistance determination and were included in resistance-specific analyses (MRSA *n* = 1395; MSSA *n* = 5214) (Figure 1).

**Figure 2:**
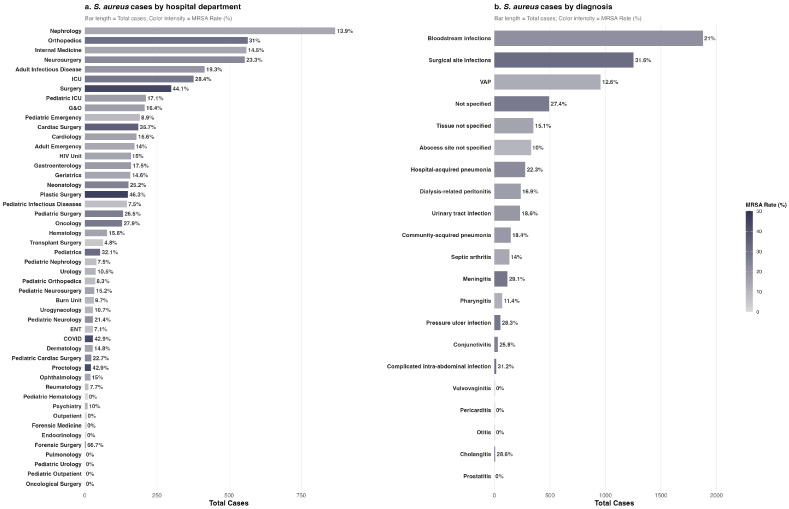
Distribution of *S. aureus* clinical isolates by hospital department (**a**) and clinical diagnosis (**b**), Hospital Civil Fray Antonio Alcalde, 2016–2025. Bar length represents total case count; color intensity indicates MRSA rate (%).

Among the 6609 isolates with resistance data, 1395 (21.1%) were classified as MRSA and 5214 (78.9%) as MSSA. Males accounted for 74.4% of isolates (*n* = 4917) and females for 25.6% (*n* = 1692), with MRSA significantly more prevalent among males (22.7% vs. 16.5%; *p* < 0.001). Isolates were obtained across 48 clinical departments, with the highest volumes in Nephrology (13.1%), Orthopedics (8.5%), Internal Medicine (8.5%), and Neurosurgery (8.4%) (Figure 2).

Clinically, bloodstream infections comprised 28.3% of cases (*n* = 1873), followed by surgical site infections (18.9%; *n* = 1248) and ventilator-associated pneumonia (14.4%; *n* = 955). Blood cultures accounted for 20.1% of specimens (*n* = 1330), followed by bronchial aspirates (9.3%; *n* = 616) and surgical wound secretion cultures (8.8%; *n* = 579). A detailed breakdown of departmental distribution, specimen source, and clinical diagnosis stratified by sex is provided in Table 1.

**Figure 3:**
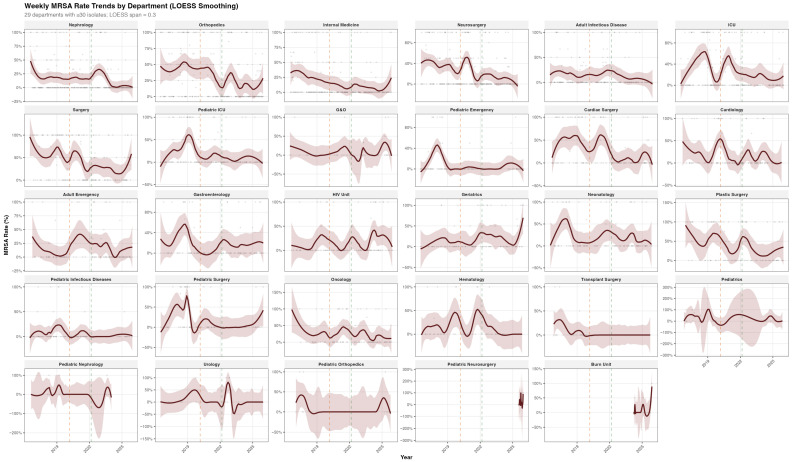
Weekly MRSA rate trends by hospital department (LOESS smoothing, span = 0.3), 2016–2025. Points indicate observed weekly MRSA rates; the solid line indicates the LOESS-smoothed trend; the shaded band denotes the 95% confidence interval. Departments with fewer than 20 total isolates were excluded (*n* = 29 departments shown). Vertical dashed lines indicate COVID-19 onset (March 2020) and post-peak transition (March 2022).

Weekly MRSA proportions were computed for each of the 48 clinical departments, yielding 3679 department-week observations and an overall mean MRSA proportion of 20.9%. Marked interdepartmental variability was observed: surgical specialties exhibited the highest MRSA burden, notably Plastic Surgery (46.3%), General Surgery (44.1%), and Cardiac Surgery (35.7%), whereas Transplant Surgery (4.8%), ENT (7.1%), and Pediatric Infectious Diseases (7.5%) had the lowest rates among departments with at least 20 isolates. LOESS smoothing confirmed that these interdepartmental disparities persisted throughout the study period (Figure 3).

**Figure 4:**
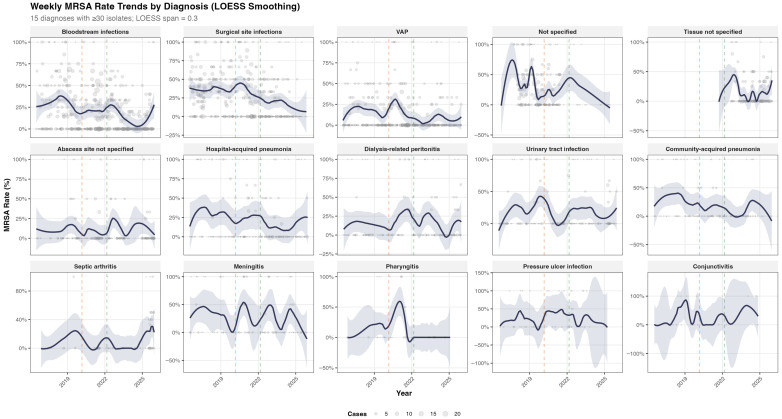
Weekly MRSA rate trends by clinical diagnosis (LOESS smoothing, span = 0.3), 2016–2025. Diagnoses with fewer than 30 total isolates were excluded (*n* = 15 diagnoses shown). Point size is proportional to weekly case count. The solid line shows the LOESS-smoothed trend and the shaded band denotes the 95% confidence interval. Vertical dashed lines indicate COVID-19 onset (March 2020) and post-peak transition (March 2022).

Weekly MRSA proportions were similarly analyzed across 21 clinical syndromes, totaling 2665 diagnosis–week observations. As illustrated in Figure 4, MRSA rates varied substantially by diagnosis. Bloodstream infections (21.0%) and surgical site infections (31.6%) showed a sustained decline over the study period. Meningitis (29.1%) and pressure ulcer infections (28.3%) exhibited intermediate resistance levels, whereas ventilator-associated pneumonia (12.6%) and abscess-site infections (10.0%) consistently demonstrated lower MRSA rates.

### 2.2. Temporal Trend Assessment of MRSA

**Figure 5:**
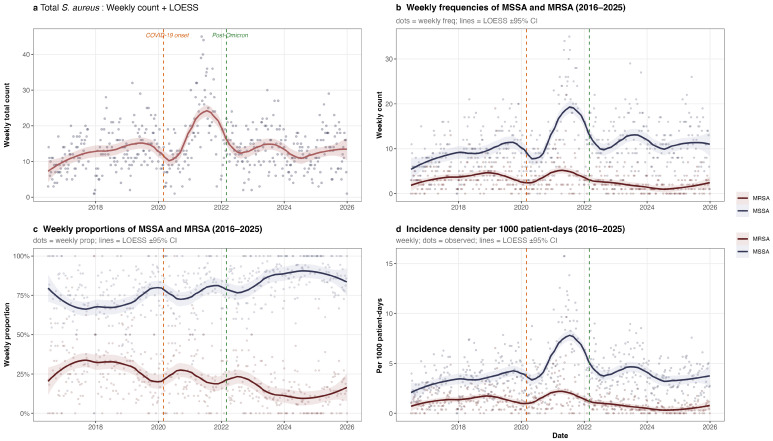
Temporal trends in clinical *Staphylococcus aureus* and MRSA isolates, Hospital Civil “Fray Antonio Alcalde”, 2016–2025. (**a**) Total weekly *S. aureus* isolate count with LOESS smoothing (span = 0.3). (**b**) Weekly MRSA and MSSA frequencies. (**c**) Weekly MRSA and MSSA proportions. (**d**) Weekly incidence density per 1000 patient-days. In all panels, dots represent observed weekly values, solid lines indicate LOESS-smoothed trends, and shaded ribbons denote 95% confidence intervals. Dashed vertical lines indicate COVID-19 onset (March 2020, orange) and post-Omicron transition (March 2022, green). The MRSA proportion declined steadily from approximately 25% pre-pandemic to below 15% post-peak (**c**), while MSSA incidence density peaked during the high-circulation phase (**d**).

The weekly proportion of MRSA declined steadily over the study period (Figure 5). LOESS smoothing revealed an early peak of approximately 33.5% in late 2017, followed by a gradual descent interrupted by a modest fluctuation during the COVID-19 surge in 2020–2021, and then a persistent downward trajectory reaching approximately 9.5% by mid-2024.

Notably, while the COVID-19 pandemic period (2020–2022) was associated with a transient increase in the absolute number of *S. aureus* infections, this surge was not mirrored in MRSA rates. As shown in Figure 5b, weekly MRSA counts remained relatively stable, whereas MSSA exhibited a marked rise. These observations suggest that the pandemic-related increase in *S. aureus* cases was driven predominantly by MSSA, consistent with a potential shift in strain dynamics or risk profiles during this period.

Formal trend analyses (Table 2) confirmed the declining MRSA trend through complementary approaches. The non-parametric Mann–Kendall test yielded a highly significant monotonic decrease (*z* = −9.03, τ = −0.282, *p* < 0.001). The Theil–Sen estimator provided a robust median weekly slope of −4.61 × 10^−4^ (95% CI: −5.60 × 10^−4^ to −3.64 × 10^−4^), corresponding to an approximate annual decline of 2.4 percentage points (95% CI: 1.9–2.9 pp/year).

To model the MRSA proportion while respecting its bounded nature, a binomial generalized linear model (GLM) with a logit link was fitted, treating weekly MRSA counts as binomial outcomes out of total *S. aureus* tested. The estimated odds ratio (OR) per year was 0.85 (95% CI: 0.829–0.871; *p* < 0.001), indicating that the odds of MRSA decreased by approximately 15% per year over the study period. The corresponding marginal effect estimated an annual decline of approximately 2.8 percentage points, consistent with the non-parametric estimates. Collectively, these results provide robust and complementary evidence that the MRSA proportion in our setting has markedly decreased over the study period.

#### Exposure-Adjusted Incidence Density Trends

**Figure 6:**
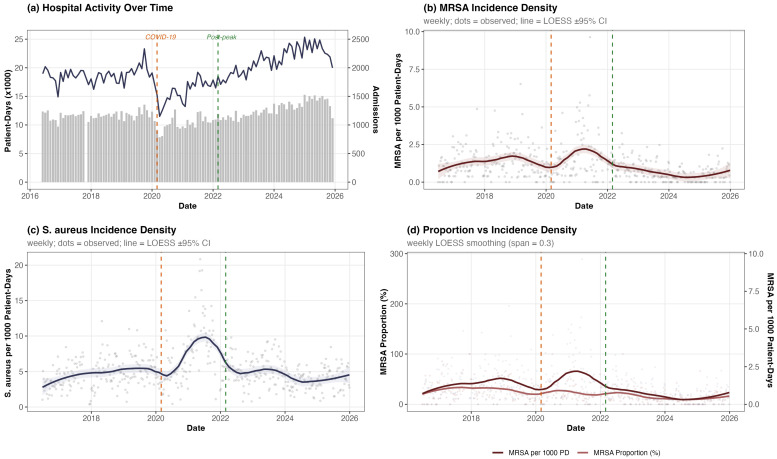
Hospital activity and infection incidence density, 2016–2025. (**a**) Monthly hospital admissions (line) and patient-days (bars). (**b**) Weekly MRSA incidence density per 1000 patient-days. (**c**) Weekly *S. aureus* incidence density per 1000 patient-days. (**d**) Comparison of weekly MRSA proportion (%) and incidence density per 1000 patient-days (dual axis). Dots indicate observed weekly values; solid lines show LOESS-smoothed trends (span = 0.3). The blue and incarnadine shaded bands represent the 95% confidence intervals around the LOESS fits.

To verify that the observed MRSA decline was not an artifact of fluctuations in hospital activity, particularly during the COVID-19 pandemic, we computed monthly incidence densities using hospitalization data as denominators (1,350,540 total patient-days; 218,303 admissions over the study period). The overall MRSA incidence density was 1.03 per 1000 patient-days (Figure 6b–d). Incidence per 1000 admissions showed concordant patterns (Supplementary Table S1 and Figure S1).

The Mann–Kendall test applied to the monthly MRSA incidence density series confirmed a significant declining trend (*z* = −4.91, τ = −0.312, *p* < 0.001), with a Theil–Sen estimated decline of 0.099 per 1000 patient-days per year (95% CI: 0.063–0.135). Importantly, the aggregate *S. aureus* incidence density (4.89 per 1000 patient-days) showed no significant temporal trend (*z* = −0.17, *p* = 0.868), indicating that the overall rate of *S. aureus* clinical isolates remained stable when adjusted for hospital volume, while the proportion attributable to MRSA decreased selectively.

The MRSA incidence density varied substantially across COVID-19 phases (Table 3). During the pre-pandemic period (2016–2020), the MRSA incidence density was 1.27 per 1000 patient-days. This increased by 19% to 1.51 per 1000 patient-days during the high-circulation period (2020–2022), coinciding with reduced hospital admissions and a higher acuity case-mix. In the post-peak period (2022–2025), MRSA incidence density fell to 0.63 per 1000 patient-days, a 50% reduction relative to the pre-pandemic baseline and a 58% reduction relative to the high-circulation period. In contrast, the *S. aureus* incidence density showed a transient surge during the high-circulation period (6.63 per 1000 patient-days) before returning to near-baseline levels post-peak (4.49 per 1000 patient-days), confirming that the pandemic-era increase was driven predominantly by methicillin-susceptible strains.

Collectively, the proportion-based and exposure-adjusted analyses provide convergent evidence that MRSA has declined markedly over the study period, with the steepest reduction occurring after the Omicron-driven pandemic peak.

### 2.3. Seasonality

Having established a declining trend in MRSA through complementary proportion-based and exposure-adjusted analyses, we next examined whether *S. aureus* and MRSA isolation rates exhibit seasonal periodicity.

#### 2.3.1. Seasonal-Trend Decomposition

**Figure 7:**
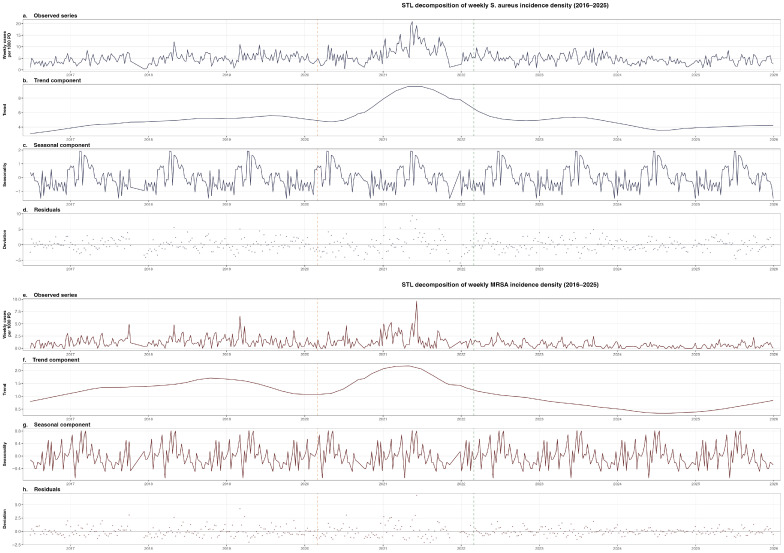
Seasonal-Trend decomposition by Loess (STL) of weekly *S. aureus* (**a**–**d**) and MRSA (**e**–**h**) incidence density per 1000 patient-days, 2016–2025. For each organism: observed series, extracted trend, seasonal component, and remainder. Dashed vertical lines indicate COVID-19 onset (March 2020, orange) and post-peak transition (March 2022, green).

STL decomposition of the weekly *S. aureus* incidence density series (per 1000 patient-days) revealed a pronounced trend component that increased from approximately 2.7 per 1000 patient-days at the beginning of the study to a peak of approximately 8.4 in mid-2021, coinciding with the height of COVID-19 hospital activity, before declining to approximately 3.5 by late 2025 (Figure 7a–d). The seasonal component exhibited a stable, annually recurring oscillation with peak-to-trough amplitude of approximately 3.2 per 1000 patient-days, with peaks consistently aligned to May–June and troughs during December–January.

For MRSA (Figure 7e–h), the trend component showed a maximum of approximately 1.9 per 1000 patient-days in mid-2021, followed by a sustained decline to a nadir of approximately 0.3 in mid-2024, mirroring the secular decline documented in Section 2.2. The trend subsequently showed a modest recovery to approximately 0.7 by late 2025. The seasonal component was present but notably weaker in absolute terms. The remainder component exhibited substantial week-to-week variability, consistent with the stochastic nature of low-frequency MRSA isolations.

#### 2.3.2. Semiparametric Seasonal Models

**Figure 8:**
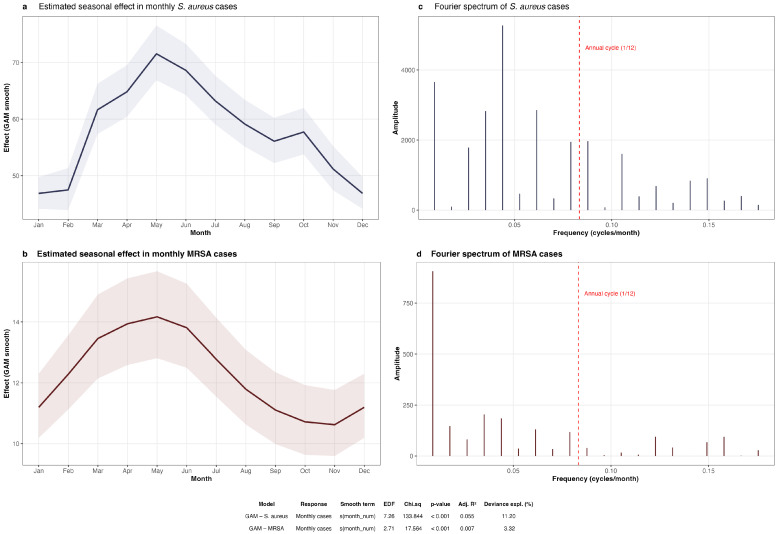
Seasonality analysis of monthly *S. aureus* and MRSA clinical isolate counts, Hospital Civil “Fray Antonio Alcalde”, 2016–2025. (**a**,**b**) Estimated seasonal effects from generalized additive models (GAM) with cyclic cubic regression splines (Poisson family, REML estimation) shown on the response scale (expected monthly counts); shaded ribbons indicate 95% confidence intervals. The seasonal smooth was statistically significant for both *S. aureus* (edf = 7.26, *p* < 0.001) and MRSA (edf = 2.71, *p* < 0.001). *S. aureus* exhibited highest predicted counts in May–June and lowest in December–January, whereas MRSA showed a concordant but attenuated peak in April–May and a nadir in October–November. (**c**,**d**) Fourier spectral power density (periodogram); the dashed red line marks the annual reference frequency (1/12 cycles per month). A prominent low-frequency component was observed for *S. aureus* (approximately 22.8-month period), while MRSA spectra were dominated by long-period variation (approximately 114 months), consistent with secular trend effects. Summary statistics are reported in the bottom table.

Generalized additive models (GAM) with cyclic cubic splines confirmed the seasonal structure identified by STL (Figure 8a,b). For *S. aureus*, the smooth effect of calendar month was highly significant (*p* < 0.001, edf = 7.26, R^2^ = 0.055), with the highest predicted counts in May–July and the lowest in December–February. The relatively high effective degrees of freedom (edf = 7.26 out of a maximum of 11) indicated a complex, non-sinusoidal seasonal shape rather than a simple harmonic oscillation.

For MRSA, the smooth term was also statistically significant (*p* < 0.001, edf = 2.71), though the proportion of deviance explained was near zero (R^2^ = 0.007). The predicted seasonal curve exhibited a modest peak in April–May and a nadir in October–November (Figure 8b), a pattern concordant with but markedly attenuated relative to aggregate *S. aureus* (amplitude of 3.5 vs. 24.7 expected cases). The near-zero R^2^ indicates that calendar month contributes negligibly to MRSA count variation once the secular declining trend is accounted for.

#### 2.3.3. Fourier Spectral Analysis

Fourier decomposition of the monthly *S. aureus* series identified a dominant spectral peak at a period of approximately 23 months, with secondary peaks near 16 and 12 months (Figure 8c). The 12-month harmonic (spectral power = 1968) confirmed the presence of an annual cycle, while the dominant quasi-biennial peak (power = 5270) and the 16-month peak (power = 2855) suggest that the seasonal dynamics of *S. aureus* isolation operate on multiple overlapping time scales, consistent with the complex seasonal shape captured by the GAM (edf = 7.26).

In contrast, the MRSA Fourier spectrum (Figure 8d) was dominated by very long periods (114 months, corresponding to the full study span), reflecting the prevailing secular decline rather than any recurring cycle. The spectral power at the 12-month frequency was only 40, approximately 50 times lower than the corresponding value for *S. aureus*, confirming a minimal annual seasonal signal.

Collectively, these results indicate that aggregate *S. aureus* isolation displays a moderate, recurring seasonal pattern peaking in late spring, whereas MRSA dynamics are governed primarily by secular trends with only marginal seasonal variation.

### 2.4. Interrupted Time Series Analysis

To formally evaluate whether the COVID-19 pandemic was associated with discrete changes in *S. aureus* and MRSA trajectories beyond the secular trends documented above, we applied a segmented regression framework with three epidemiologically defined phases. Both *S. aureus* and MRSA models used weekly aggregation (476 observations) to maintain analytical symmetry and maximize statistical power.

#### 2.4.1. *S. aureus* Weekly Counts

The Durbin–Watson test on OLS residuals detected significant positive autocorrelation in the weekly *S. aureus* series (DW = 1.225, *p* < 0.001), motivating the use of GLS-AR(1) and Newey–West corrections.

In the GLS-AR(1) model (AR(1) phi = 0.386), the pre-pandemic baseline showed a modest but significant upward trend of +0.037 isolates per week (*p* = 0.005), consistent with the gradual increase in total *S. aureus* isolations observed in the LOESS analysis (Section 2.2). The onset of the high-circulation phase was associated with an immediate level drop of −6.0 isolates per week (*p* = 0.009), followed by a significant acceleration in the weekly rate of change (+0.122 isolates per week, *p* < 0.001), yielding a net high-circulation slope of +0.158 isolates per week. This accelerated accumulation during 2020–2022 is consistent with the surge in MSSA-driven *S. aureus* cases documented in Figure 5b.

At the transition to the post-peak period, a further level shift of −11.0 isolates per week occurred (*p* < 0.001), accompanied by a significant slope reversal of −0.166 isolates per week (*p* < 0.001), resulting in a near-zero net post-peak slope of −0.008 isolates per week. These findings were consistent across all three estimation approaches (Table 4). The joint Wald *F*-test for the cumulative slope change was highly significant (*F* = 14.7, *p* < 0.001), confirming that the post-pandemic *S. aureus* trajectory differs markedly from the pre-pandemic trend.

**Table 4:** Interrupted time series regression coefficients for weekly *S. aureus* and weekly MRSA counts across three estimation methods, 2016–2025.

Coefficient	Description	*S. aureus* OLS	*S. aureus* GLS-AR(1)	*S. aureus* NW	MRSA OLS	MRSA GLS-AR(1)	MRSA NW
β_0_	Intercept	9.23 ***	9.26 ***	9.23 ***	3.06 ***	3.07 ***	3.06 ***
β_1_	Pre-pandemic slope	0.038 ***	0.037 **	0.038 ***	0.006	0.005	0.006
β_2_	High-circ level shift	−6.67 ***	−5.98 **	−6.67 **	−0.94	−0.78	−0.94
β_3_	High-circ slope change	0.128 ***	0.122 ***	0.128 *	0.012	0.01	0.012
β_4_	Post-peak level shift	−11.36 ***	−10.99 ***	−11.36 **	−2.32 ***	−2.21 **	−2.32 *
β_5_	Post-peak slope change	−0.173 ***	−0.166 ***	−0.173 **	−0.023 *	−0.021	−0.023
	AR(1) phi	–	0.386	–	–	0.242	–
	Durbin-Watson	1.225 ***	–	–	1.546 ***	–	–
	Joint test ($H_{0}:\;\beta_{3}+\beta_{5}=0$)	*F* = 14.7 ***	–	–	*F* = 5.5 *	–	–

Both models use weekly counts (476 observations). Phase boundaries: high viral circulation from March 2020; post-peak stabilization from March 2022. β_1_ represents the baseline (pre-pandemic) slope; β_2_ and β_4_ capture immediate level shifts at each transition; β_3_ and β_5_ capture changes in slope relative to the preceding phase. Net slope within each phase is obtained by cumulative addition (e.g., post-peak net slope = β_1_ + β_3_ + β_5_ see Table 5). * *p* < 0.05; ** *p* < 0.01; *** *p* < 0.001. OLS, ordinary least squares; GLS-AR(1), generalized least squares with first-order autoregressive correlation; NW, Newey–West heteroscedasticity- and autocorrelation-consistent standard errors. Joint test evaluates ($H_{0}:\;\beta_{3}+\beta_{5}=0$) (OLS model). A sensitivity analysis using monthly MRSA aggregation (114 observations) yielded concordant results (joint Wald *F* = 4.0, *p* = 0.048), see Supplementary Table S2.

**Table 5:** Net slopes by COVID-19 phase from GLS-AR(1) models.

Outcome	Pre-Pandemic	High Circulation	Post-Peak	Joint Test *p*-Value
*S. aureus* counts (per week)	+0.037	+0.158	−0.008	<0.001
MRSA counts (per week)	+0.005	+0.015	−0.006	0.019
MRSA incidence density (per 1000 PD/week)	+0.002	+0.004	−0.003	0.012

Note: Net slope = sum of baseline slope and all applicable slope-change coefficients for each phase. Joint test evaluates ($H_{0}:\;\beta_{3}+\beta_{5}=0$) (OLS model). PD, patient-days. A sensitivity analysis using monthly MRSA aggregation yielded concordant joint Wald results (*p* = 0.048 for counts; *p* = 0.044 for incidence density).

#### 2.4.2. MRSA Weekly Counts

The Durbin–Watson test on the weekly MRSA count series also indicated significant positive autocorrelation (DW = 1.546, *p* < 0.001). In the GLS-AR(1) model (AR(1) phi = 0.242), the pre-pandemic baseline slope was +0.005 cases per week (*p* = 0.261), consistent with a non-significant upward drift. Neither the high-circulation level shift (−0.78; *p* = 0.357) nor the associated slope change (+0.010; *p* = 0.464) reached statistical significance, indicating that the pandemic onset did not produce a detectable abrupt change in weekly MRSA counts.

In contrast, the transition to the post-peak period was marked by a significant immediate level drop of −2.21 MRSA cases per week (*p* = 0.008), representing an abrupt reduction of approximately 2 cases per week following the Omicron-driven pandemic peak. The post-peak slope change was −0.021 cases per week (*p* = 0.107), which, while not individually significant, contributed to a net post-peak slope of −0.006 cases per week (downward trajectory). Results were consistent across all three estimation approaches (Table 4).

The formal joint Wald *F*-test evaluating whether the cumulative slope change across both post-pandemic phases differed from zero ($H_{0}:\;\beta_{3}+\beta_{5}=0$) was significant (*F* = 5.5, *p* = 0.019), confirming that the combined shift in the MRSA trajectory was statistically distinguishable from the pre-pandemic baseline. As a sensitivity analysis, the same ITS model was applied to monthly MRSA counts (114 observations), yielding qualitatively similar results: the joint Wald test remained significant (*F* = 4.0, *p* = 0.048), although no individual coefficient reached significance, reflecting the reduced statistical power of the monthly aggregation.

#### 2.4.3. Incidence Density ITS

To verify that the observed ITS patterns were not confounded by changes in hospital volume during the pandemic, the segmented regression framework was applied to weekly MRSA incidence density per 1000 patient-days (476 observations). Results were broadly consistent with the count-based analysis. In the GLS-AR(1) model (phi = 0.268), the post-peak level shift was −0.84 per 1000 patient-days (*p* = 0.011), confirming that the abrupt post-Omicron reduction in MRSA persisted after adjustment for fluctuating denominators. The post-peak slope change was −0.007 per 1000 patient-days per week (*p* = 0.222). The joint Wald test for the cumulative slope change was significant (*F* = 6.4, *p* = 0.012). Monthly aggregation as a sensitivity analysis yielded concordant results (*F* = 4.2, *p* = 0.044).

For aggregate *S. aureus* incidence density, the OLS model revealed a significant post-peak level shift (−2.4 per 1000 patient-days, *p* = 0.021) and slope change (−0.157, *p* = 0.019), indicating that the reduction in *S. aureus* clinical isolates persisted even after adjustment for hospital activity. A summary of net slopes by phase is presented in Table 5.

## 3. Discussion

Over a 9.5-year surveillance period encompassing the pre-pandemic, COVID-19, and post-pandemic eras, we documented a sustained and statistically significant decline in MRSA among *S. aureus* clinical isolates at a large Mexican tertiary-care hospital. The MRSA proportion decreased from 28.1% during the pre-pandemic period (2016–2020) to 14.0% in the post-peak period (2022–2025), with LOESS smoothing revealing a peak of approximately 33.5% in late 2017, followed by a persistent downward trajectory reaching approximately 9.5% by mid-2024. The non-parametric Mann–Kendall test confirmed this monotonic decrease (*z* = −9.03, tau = −0.282, *p* < 0.001), and the Theil–Sen estimator yielded a median weekly reduction of 4.61 × 10^−4^ in MRSA proportion, corresponding to an annual decline of 2.4 percentage points (95% CI: 1.9–2.9). A binomial GLM with logit link estimated an odds ratio of 0.85 per year (95% CI: 0.829–0.871; *p* < 0.001), indicating that the odds of MRSA decreased by approximately 15% annually over the study period.

Crucially, when adjusted for hospital activity using patient-days denominators, the MRSA incidence density declined from 1.27 to 0.63 per 1000 patient-days, a 50% reduction, while the aggregate *S. aureus* incidence density remained stable (4.89 per 1000 patient-days; Mann–Kendall *z* = −0.17, *p* = 0.868). This dissociation confirms that the MRSA decline represents a selective reduction in resistant strains rather than an artifact of fluctuating hospital volume. Interrupted time series analysis demonstrated that the cumulative post-pandemic shift in MRSA trajectory was statistically significant (joint Wald test: *F* = 5.5, *p* = 0.019 for counts, *F* = 6.4, *p* = 0.012 for incidence density), with the steepest decline occurring after the Omicron-driven pandemic peak in early 2022.

Weekly case counts also exhibited a recurring seasonal pattern. Total *S. aureus* isolations increased predictably from April through July each year, forming a broad mid-year peak confirmed by GAM analysis (*p* < 0.001, edf = 7.26). A seasonal signal was also statistically detectable for MRSA (*p* < 0.001, edf = 2.71), although the proportion of deviance explained was negligible (R^2^ = 0.007), indicating that MRSA dynamics are governed primarily by secular trends rather than seasonal forcing.

The pandemic timeline revealed a complex perturbation in *S. aureus* dynamics. During the period of high viral circulation (March 2020 to February 2022), overall *S. aureus* incidence increased—primarily driven by methicillin-susceptible strains—with the *S. aureus* incidence density rising transiently to 6.63 per 1000 patient-days. Notably, MRSA did not exhibit the surge reported in several high-income settings [20,22,32,33]. Instead, the MRSA incidence density increased modestly to 1.51 per 1000 patient-days during the high-circulation period before declining sharply to 0.63 per 1000 patient-days post-peak. In the weekly ITS analysis, the post-peak level shift was individually significant (*p* = 0.008, GLS-AR(1)), and the joint Wald test confirmed that the combined shift in trajectory was distinguishable from the pre-pandemic baseline (*p* = 0.019). A sensitivity analysis using monthly aggregation (114 observations) yielded concordant results (joint *p* = 0.048), though with reduced power for individual coefficients. These findings suggest that pandemic-related pressures did not reverse, and may have coincided with an acceleration of the pre-existing downward trend.

These aggregate trends masked substantial clinical heterogeneity. At the departmental level, MRSA accounted for 44.1% of isolates in General Surgery compared with 13.9% in Nephrology. At the syndromic level, MRSA prevalence reached 31.6% in surgical site infections and 21.0% in bloodstream infections—the two most frequently observed infectious syndromes. Throughout the study period, male patients contributed the majority of cases (74.2%) and consistently exhibited higher resistance rates than females (22.7% vs. 16.5%; *p* < 0.001). Together, these findings depict a hospital where MRSA is broadly in decline yet persists within specific high-risk departments and clinical syndromes, necessitating targeted infection-control strategies even as the overall trend remains favorable.

Published reports indicate that during 2020–2021 many institutions experienced divergent trends: some documented reductions of 28–41% associated with intensified hand hygiene and PPE use, while others reported increases in MRSA detection, reflecting heterogeneous pandemic impacts [20,22,32,34]. In Europe, declining MRSA trends have been documented in Germany [22] and Spain [35], attributed to national infection prevention campaigns and antimicrobial stewardship programs. In contrast, data from Latin America remain comparatively scarce. The SENTRY Antimicrobial Surveillance Program reported MRSA rates of 40–50% in Latin American hospitals during 2010–2015, substantially higher than concurrent rates in North America and Europe [36]. Few single-center studies from the region have provided the longitudinal resolution needed to characterize temporal dynamics with the precision attempted here [37]. Our findings, documenting a decline from approximately 28% to 14%, suggest that the global downward trajectory in MRSA prevalence extends to the Latin American tertiary-care setting, albeit from a higher baseline.

The segmented regression model employed two transition points defining three epidemiologically distinct phases. The first transition, placed at March 2020, corresponds to the identification of the first confirmed COVID-19 cases in the state of Jalisco on March 14, 2020, the World Health Organization’s declaration of a global pandemic on March 11, 2020, and the subsequent implementation of social distancing measures, including suspension of elective surgeries and hospital reorganization at our institution [38].

The second transition, placed at March 2022, marks the post-Omicron stabilization period. By this date, the Omicron variant wave had peaked in Mexico (January–February 2022), national COVID-19 vaccination coverage had exceeded 70% of the adult population with at least one dose [39], and our institution had progressively resumed elective surgical services and pre-pandemic operational capacity. Hospital admissions, which had declined by 12% during the high-circulation period, returned to and exceeded pre-pandemic levels during the post-peak period (Table 3). These operational milestones, combined with the epidemiological trajectory of COVID-19 in our region, provide a rational basis for the selected phase boundaries. Sensitivity analyses using alternative cutpoints yielded qualitatively similar results, supporting the robustness of the segmented model.

The magnitude of the MRSA decline carries direct implications for clinical practice. The halving of MRSA proportion from 28.1% to 14.0%, and the parallel 50% reduction in MRSA incidence density (from 1.27 to 0.63 per 1000 patient-days), represent a substantial shift in the epidemiological landscape of staphylococcal infections at our institution.

From a therapeutic perspective, empiric antibiotic selection for suspected staphylococcal infections is guided by local MRSA prevalence thresholds. Clinical practice guidelines recommend empiric anti-MRSA coverage (e.g., vancomycin or linezolid) when the institutional MRSA rate exceeds 10–20%, depending on the clinical syndrome and patient risk factors [2,40]. With MRSA proportions now approaching 14% overall and falling below 13% in several clinical syndromes (e.g., ventilator-associated pneumonia at 12.6%, abscess infections at 10.0%), the continued reflexive use of anti-MRSA agents for all suspected staphylococcal infections warrants reassessment. Reductions in unnecessary vancomycin use could mitigate selection pressure for vancomycin-resistant organisms, decrease nephrotoxicity, and reduce healthcare costs.

For antimicrobial stewardship programs, our data provide evidence-based support for periodic review of empiric therapy guidelines based on institutional resistance trends [41,42]. The convergence of proportion-based and exposure-adjusted analyses strengthens the case for de-escalation protocols that account for evolving resistance epidemiology.

The progressive decline of MRSA is likely multifactorial, involving both epidemiological and microbiological mechanisms. A primary driver is the implementation and intensification of infection control measures in healthcare settings [34,43]. These include improved hand hygiene, contact precautions, active surveillance, decolonization protocols, and enhanced environmental cleaning [34,43]. Such interventions have been temporally associated with marked reductions in hospital-onset MRSA infections, particularly in intensive care units, and have been shown to reduce transmission of healthcare-associated MRSA clones such as USA100 in the United States and ST228-I in Europe [34,35,43,44].

Clonal replacement is another important mechanism. Over time, certain epidemic MRSA clones have been supplanted by others with different fitness characteristics [44,45,46]. In several European and North American hospitals, older clones (e.g., ST228-I, CC45-MRSA-IV) have been replaced by more successful clones (e.g., CC22-MRSA-IV, CC5-MRSA-II, CC8-IV), which may have altered virulence, transmissibility, or antimicrobial susceptibility profiles [35,44,45]. Some of these newer clones exhibit lower levels of antimicrobial resistance, possibly reflecting a fitness advantage in the absence of strong antibiotic selection pressure [46,47]

Microevolutionary changes within MRSA lineages may also play a role. There is evidence that the maintenance of methicillin resistance, conferred by the *mecA* gene, imposes a fitness cost on *S. aureus* in the absence of selective antibiotic pressure [48,49]. Loss of resistance determinants (e.g., mecA or SCCmec elements) has been observed in certain lineages, leading to the re-emergence of methicillin-susceptible *S. aureus* (MSSA) from previously resistant backgrounds, particularly when the selective advantage of resistance diminishes due to reduced antibiotic use or effective infection control [48].

The absence of a pronounced MRSA spike may reflect the influence of several mitigating factors. Broad antibiotic de-escalation guidelines implemented during the pandemic likely helped limit unnecessary use of broad-spectrum antibiotics [2,42]. Additionally, the early adoption of SARS-CoV-2–specific therapeutics may have reduced empiric antibiotic prescribing [41]. Continued MRSA admission screening for patients with severe pneumonia, alongside the sustained implementation of contact precautions, may also have contributed to the stable MRSA proportion during this period [42,50]. This combination of measures likely preserved previous gains in antimicrobial resistance control and facilitated the accelerated decline in MRSA incidence observed after 2022.

Several factors beyond secular trends and pandemic effects could have influenced the observed changes and merit consideration. Changes in clinical sampling practices over the study period—such as shifts in the indications for obtaining cultures or the types of specimens collected—could affect the measured MRSA rate independently of true prevalence changes. Similarly, referral pattern modifications during the pandemic, with preferential admission of more severely ill patients during high-circulation periods, could have altered the case-mix and the underlying probability of MRSA isolation.

The absence of detailed antibiogram policy data prevents us from disentangling the specific contributions of stewardship interventions from broader temporal trends. Formulary changes, such as the introduction or restriction of specific antibiotics, could alter MRSA selection pressure. Additionally, changes in hospital infrastructure, staffing ratios, and infection control staffing levels over the study period were not captured in our analysis. Patient-level confounders—including comorbidity burden, prior antibiotic exposure, prior healthcare contact, and duration of hospitalization before culture collection—were not available in our dataset and represent important unmeasured sources of confounding.

The ecological nature of the analysis, which relies on aggregate time-series data rather than patient-level outcomes, precludes definitive causal attribution of the MRSA decline to any specific intervention or exposure.

The statistical framework employed in this study was designed to address specific methodological concerns. Multiple analytical approaches were applied to the same outcomes—including non-parametric tests (Mann–Kendall), robust regression (Theil–Sen), parametric models (binomial GLM), and time-series methods (ITS with OLS, GLS-AR(1), and Newey–West corrections)—to test the same pre-specified primary hypothesis of a declining MRSA trend, rather than to screen multiple independent outcomes. Accordingly, formal adjustment for multiple comparisons (e.g., Bonferroni or false discovery rate correction) was not applied, consistent with established recommendations against such adjustments when testing pre-specified hypotheses with converging methods [51]. The convergence of all analytical approaches toward the same conclusion provides strong evidence against a type I error explanation. Methodological strengths include analytical symmetry achieved by applying weekly aggregation to both *S. aureus* and MRSA ITS models (476 observations each), maximizing comparability and statistical power; monthly aggregation for MRSA was retained only as a sensitivity analysis.

For the ITS analysis, the primary weekly model (476 observations) detected a significant post-peak level shift for MRSA (*p* = 0.008, GLS-AR(1)), and the joint Wald test for the cumulative slope change (*H*_0_: β_3_ + β_5_ = 0) was significant (*p* = 0.019 for counts; *p* = 0.012 for incidence density). The individual post-peak slope change coefficient was non-significant (*p* = 0.107, GLS-AR(1)), consistent with a gradual rather than abrupt change in the rate of decline. A sensitivity analysis using monthly aggregation (114 observations) yielded concordant joint Wald results (*p* = 0.048 counts; *p* = 0.044 incidence density), though no individual coefficient reached significance, confirming that the reduced power of the monthly resolution—rather than absence of an effect—accounted for the non-significance.

This study has several limitations. First, as a single-center study conducted at a large tertiary-care hospital in western Mexico, the generalizability of our findings to other healthcare settings—including smaller hospitals, community settings, or other geographic regions—is uncertain. Tertiary-care hospitals receive referrals of complex cases and may have MRSA epidemiology that differs from community or primary-care settings. Second, molecular typing data (e.g., *spa* typing, multilocus sequence typing, whole-genome sequencing) were not available, precluding assessment of clonal dynamics, lineage displacement, or the relative contributions of healthcare-associated versus community-associated MRSA strains. Such data would be essential to understand the biological mechanisms underlying the observed decline. Third, detailed data on antimicrobial stewardship policies, formulary changes, and infection control interventions implemented during the study period were not systematically recorded. This limits our ability to attribute the MRSA decline to specific programmatic interventions. Fourth, hospital-level denominators were approximated using the LOS-sum method, which aggregates the total length of stay of admissions within each time period. While this approach provides a reasonable estimate of patient-days at risk, it may not perfectly capture real-time census fluctuations, particularly during the pandemic when short-stay and ICU admissions varied substantially. The concordance between count-based and incidence density-based analyses mitigates this concern. Fifth, the analysis is ecological in nature, based on aggregate counts rather than patient-level data. We cannot exclude the ecological fallacy: institutional-level trends may not reflect changes in individual-level risk. Similarly, we were unable to adjust for patient-level confounders such as prior antibiotic exposure, comorbidities, immunosuppression, or prior healthcare contact. Sixth, isolates lacking susceptibility data (4.6%) were excluded from MRSA/MSSA stratification. While the proportion is small, non-random missingness could introduce bias if the probability of missing resistance data correlates with MRSA status. Seventh, although our institution maintained cefoxitin-based MRSA screening per CLSI M100 guidelines throughout the study period, minor updates to CLSI interpretive breakpoints, changes in laboratory instrumentation (including transitions between automated susceptibility platforms), or variations in testing protocols over the 9.5-year period could theoretically affect MRSA detection rates independently of true prevalence changes. The consistency of our findings across multiple analytical approaches mitigates this concern. Eighth, the dataset encompasses all clinical *S. aureus* isolates, including both community-onset and hospital-acquired infections, without applying formal healthcare-associated infection (HAI) definitions (e.g., CDC/NHSN criteria). Trends should therefore be interpreted as reflecting the overall institutional burden of clinical *S. aureus* rather than strictly nosocomial events. Ninth, although both primary ITS models used weekly aggregation (476 time points each), the lower absolute counts of MRSA compared with total *S. aureus* still limit statistical power for detecting individual coefficient-level effects, particularly for slope changes within specific phases.

Despite these limitations, the study has notable strengths. The 9.5-year study period spanning three epidemiologically distinct phases provides a uniquely comprehensive temporal perspective. The use of multiple complementary analytical approaches with formal autocorrelation correction enhances robustness. The inclusion of exposure-adjusted incidence densities alongside proportion-based analyses directly addresses potential confounding by pandemic-related fluctuations in hospital activity. The large sample size (6609 isolates) provides sufficient statistical power for the primary analyses.

Future research should integrate whole-genome sequencing of archived isolates to elucidate clonal shifts, while a multicenter network spanning western Mexico could validate our findings across diverse settings. Incorporating antimicrobial-use density metrics into interrupted time-series frameworks [52] will quantify the precise contribution of stewardship policies, and patient-level modeling can refine risk-stratified prevention bundles.

## 4. Materials and Methods

### 4.1. Setting, Registry Consultation, and Data Extraction

This retrospective study was conducted at Antiguo Hospital Civil de Guadalajara “Fray Antonio Alcalde,” a 1000-bed tertiary referral and teaching institution affiliated with the University of Guadalajara in Jalisco, Mexico. The hospital primarily serves an uninsured population from western Mexico, providing specialized care for both adults and children.

We consulted the historical microbiology laboratory databases to identify all clinical *Staphylococcus aureus*-positive cultures collected between June 2016 and December 2025. The dataset encompasses both community-onset infections diagnosed at admission and hospital-acquired infections identified during inpatient care; no minimum length-of-stay criterion was applied. Colonization surveillance cultures (nares, axillae) and environmental samples were excluded. Using the electronic medical record system, we extracted patient demographic information, specimen source, hospital department of origin, and antibiotic-susceptibility phenotypes.

To compute exposure-adjusted incidence rates, monthly hospitalization denominators (number of admissions and patient-days) were obtained from the institutional admissions registry for the same study period. Incidence density was expressed as the number of isolates per 1000 patient-days; incidence per 1000 admissions was calculated as a secondary measure and is reported in Supplementary Table S1 and Figure S1. Microbiology laboratory records were available without interruption for the entire study period, and hospitalization denominator data were complete and validated through December 2025.

### 4.2. Patient Inclusion and Antimicrobial Susceptibility Testing

The study population comprised patients with at least one clinical specimen culture positive for *S. aureus* and a clinical syndrome compatible with active infection. Phenotypic classification as MRSA or methicillin-susceptible *S. aureus* (MSSA) was based primarily on cefoxitin screening, the recommended surrogate marker for mecA-mediated resistance per CLSI M100 guidelines [53] and CDC laboratory guidance [54]. Cefoxitin was chosen over oxacillin as the primary classifier because it is a more potent inducer of the *mecA* gene, yields clearer zone-diameter endpoints, and offers higher sensitivity for detecting heteroresistant strains [55]. Archived cefoxitin disk-diffusion zone diameters (30 µg disk, Mueller–Hinton agar, 35 °C, 16–18 h; susceptible ≥22 mm, resistant ≤21 mm) and oxacillin broth-microdilution MIC values (susceptible ≤2 µg/mL; resistant ≥4 µg/mL) were extracted from the laboratory information system. Of 6600 isolates with both cefoxitin and oxacillin results, 1020 (15.5%) showed discrepant classifications; the majority (757/1020, 74.2%) were cefoxitin-resistant but oxacillin-susceptible, a pattern consistent with the known lower sensitivity of oxacillin MIC testing for mecA-positive strains. Additional susceptibility profiles generated by the Vitek 2 automated platform (bioMérieux, Marcy-l’Étoile, France) were likewise retrieved. Quality control was verified through archived run records confirming inclusion of *S. aureus* ATCC 25923 and ATCC 29213 reference strains in each testing batch.

### 4.3. Data Management and Quality Control

Before analysis, all extracted records underwent standardized data cleaning consistent with accepted antibiogram and AMR surveillance practices. Only diagnostic clinical isolates were retained; non-clinical cultures—including colonization screening swabs (nares, axillae, rectal, inguinal, and perineal sites) and environmental samples—were excluded. To minimize overrepresentation from repeat sampling, we applied episode-based deduplication, retaining a single isolate per patient and specimen type within a ≤3-day window. MRSA status was assigned using a hierarchical algorithm: cefoxitin results (disk or MIC) were used as the primary surrogate for mecA-mediated oxacillin resistance; when cefoxitin data were unavailable, oxacillin MIC was used as a fallback (Figure 1), defining resistance as ≥4 μg/mL per CLSI M100 [53]. Isolates lacking both cefoxitin and oxacillin susceptibility data were retained for aggregate *S. aureus* counts but excluded from resistance-specific analyses. Finally, records with missing key fields required for time-series analyses were excluded from temporal models.

### 4.4. Statistical Analysis

Demographic data were summarized as simple relative frequencies. The proportion of MRSA was calculated by dividing the number of MRSA-positive isolates by the total number of isolates with a valid resistance determination for each time period. Data normality was assessed using the Shapiro–Wilk test. Categorical variables were compared using Pearson’s chi-square test or Fisher’s exact test, as appropriate. Trend and seasonality analyses were performed on weekly or monthly aggregated series, and exposure-adjusted incidence densities (per 1000 patient-days) were analyzed in parallel to account for fluctuations in hospital activity.

#### 4.4.1. Temporal Trend Assessment of MRSA

To stabilize week-to-week variation in sample size, we calculated both absolute weekly counts and their corresponding proportions for MSSA and MRSA. We then applied locally estimated scatterplot smoothing (LOESS) with a reduced span (0.3) to each series, yielding nonparametric estimates of the secular trend and its 95% confidence band. LOESS fits a low-degree polynomial within a moving neighborhood defined by the span parameter, revealing underlying trends and transient shifts without imposing rigid parametric assumptions. This visualization guided subsequent formal time-series analyses.

To formally assess monotonic trends without distributional assumptions, we applied the Mann–Kendall test to the weekly MRSA proportion series and to the monthly MRSA incidence density (per 1000 patient-days). We then used the Theil–Sen estimator to derive a robust, median-based estimate of the weekly rate of change in MRSA proportion, with nonparametric confidence intervals.

To model the MRSA proportion as a function of time while respecting the bounded nature of the outcome, we fitted a binomial generalized linear model (GLM) with a logit link, treating weekly MRSA counts as binomial outcomes (MRSA events out of total *S. aureus* tested). The model took the form:

*logit*(*p_t_*) = *log*(*p_t_*/(1 − *p_t_*)) = β_0_ + β_1_ · time*_t_*

where *p_t_* denotes the probability of MRSA at week *t*. The odds ratio per year and its 95% confidence interval were derived by exponentiating the scaled coefficient (OR_year_ = exp(52 × β_1_)). Marginal effects were computed to express the estimated annual decline in percentage points.

#### 4.4.2. Seasonality

To assess the presence of seasonal patterns in *S. aureus* and MRSA, we applied a triangulated approach combining smoothing decomposition, harmonic analysis, and semiparametric regression.

Seasonal-Trend Decomposition (STL). We applied Seasonal-Trend decomposition based on Loess (STL) to the weekly *S. aureus* and MRSA incidence density series (per 1000 patient-days) to separate long-term trends, seasonal variation, and residual components. Weekly resolution was chosen for STL to maximize temporal granularity while leveraging the exposure-adjusted denominator.

Generalized Additive Models (GAM). Monthly aggregation was used for GAM and Fourier analyses because it provides sufficient counts per bin to avoid zero-inflation and aligns with epidemiological surveillance reporting conventions. We fitted GAMs with cyclic cubic spline terms (*k* = 12) under a Poisson family to estimate the smooth effect of calendar month on case counts:

*log*(μ*_t_*) = β_0_ + *f*(month*_t_*) + β_1_ · time*_t_*

where *f*(month*_t_*) is a cyclic cubic regression spline with *k* = 12 knots. Formal hypothesis testing of seasonality was performed by evaluating the significance of the smooth term (*p*-value for *f* ≠ 0).

Fourier Spectral Analysis. We implemented Fourier analysis using the base fft() function to decompose each series into component frequencies and identify dominant periodicities. The spectral power at the 12-month (annual) frequency was extracted to quantify the strength of the yearly cycle.

Together, these methods provided both statistical and visual confirmation of seasonal structure.

#### 4.4.3. Interrupted Time Series Analysis of COVID-19-Associated Changes

We divided the study period into three epidemiologically defined phases:

- Pre-pandemic baseline (July 2016–February 2020): the pre-COVID-19 reference period.
- High viral circulation (March 2020–February 2022): encompassing the first pandemic waves through the Omicron peak, coinciding with documented reductions in hospital admissions and elective procedures.
- Post-peak stabilization (March 2022–December 2025): the transition to endemic COVID-19 transmission following the Omicron apex.

A segmented linear regression model was fitted to estimate immediate level shifts at each breakpoint and subsequent trend changes:

$$Y_{t}=\beta_{0}+\beta_{1}\cdot {\mathrm{time}}_{t}+\beta_{2}\cdot {\mathrm{high}\text{\_}\mathrm{circ}}_{t}+\beta_{3}\cdot t_{\mathrm{high},t}+\beta_{4}\cdot {\mathrm{post}\text{\_}\mathrm{peak}}_{t}+\beta_{5}\cdot t_{\mathrm{post},t}+\varepsilon_{t}$$

where

- time*_t_* is the continuous time index (weeks for *S. aureus*, months for MRSA);
- high_circ*_t_* and post_peak*_t_* are binary indicator variables for the respective phases;
- *t*_high,*t*_ and *t*_post,*t*_ capture the change in slope relative to the preceding period;
- *ε_t_* is the error term.

The net slope within each phase is obtained by summing the relevant coefficients:
**Phase****Net Slope Expression**Pre-pandemicβ_1_High circulationβ_1_ + β_3_Post-peakβ_1_ + β_3_ + β_5_

Autocorrelation correction. Because temporal autocorrelation can inflate type I error rates in segmented regression, we tested residual serial correlation with the Durbin–Watson (DW) test. Two complementary corrections were applied:

- GLS-AR(1): Generalized least squares with a first-order autoregressive correlation structure, estimated via restricted maximum likelihood (REML), which explicitly models the temporal dependence in the residuals.
- Newey–West HAC: Ordinary least squares with heteroscedasticity- and autocorrelation-consistent (HAC) standard errors, which adjusts inference without altering point estimates.

All three sets of estimates (OLS, GLS-AR(1), Newey–West) are reported to assess robustness of the findings.

Formal slope comparison. To test whether the combined post-pandemic slope change differed significantly from zero, we applied a joint Wald *F*-test on the linear hypothesis:

$$H_{0}:\;\beta_{3}+\beta_{5}=0$$

This evaluates whether the net change in trend across the high-circulation and post-peak periods jointly differs from the pre-pandemic baseline slope.

Incidence density ITS. The segmented regression framework was additionally applied to weekly *S. aureus* and MRSA incidence densities (per 1000 patient-days) to verify that observed trends were not artifacts of changes in hospital volume during the pandemic. Sensitivity analysis. To assess the robustness of the MRSA results to the choice of temporal aggregation, the ITS analysis was additionally performed using monthly MRSA counts and monthly incidence density (114 observations). Results from the monthly aggregation are reported alongside the primary weekly analysis.

#### 4.4.4. Software

All analyses were performed in R version 4.5.2 (R Foundation for Statistical Computing, Vienna, Austria). Data import was handled with readxl (v1.4.5); data wrangling and temporal aggregation with the tidyverse suite (v2.0.0), lubridate (v1.9.5), and zoo (v1.8.15). Nonparametric trend testing used the Kendall (v2.2.2) and trend (v1.1.6) packages (Mann–Kendall test) and mblm (v0.12.1; Theil–Sen estimator). The binomial GLM was fitted with stats::glm (base R). Seasonal structure was evaluated with STL decomposition via forecast (v9.0.1), generalized additive models with cyclic cubic splines via mgcv (v1.9.3), and Fourier spectral analysis using the base fft() function. Interrupted time series models were fitted via stats::lm (OLS) and nlme::gls (v3.1.168; GLS-AR(1)); Newey–West HAC standard errors were computed with sandwich (v3.1.1) and lmtest (v0.9-40). Formal slope comparisons used car::linearHypothesis (v3.1.5). Figures were composed with ggplot2 (v4.0.2) and patchwork (v1.3.2), using the viridis (v0.6.5) color palette for accessibility. All code is available upon request.

## 5. Conclusions

Over a 9.5-year surveillance period encompassing the COVID-19 pandemic, the proportion of MRSA among clinical *S. aureus* isolates at a Mexican tertiary-care hospital declined from 28.1% to 14.0%, with a corresponding 50% reduction in MRSA incidence density (1.27 to 0.63 per 1000 patient-days). This decline was confirmed by multiple complementary analytical approaches–non-parametric trend tests, binomial regression, and interrupted time series analysis with formal autocorrelation correction–and persisted after adjustment for pandemic-related fluctuations in hospital activity. While aggregate *S. aureus* isolation rates exhibited moderate seasonality, MRSA dynamics were driven predominantly by secular trends. The findings suggest that the global downward trajectory in MRSA prevalence extends to the Latin American tertiary-care setting and provide evidence-based support for periodic reassessment of empiric anti-MRSA prescribing policies. The sustained decline, coupled with the absence of a pandemic-era MRSA rebound, raises the hypothesis that successful infection-control practices may have coincided with clonal replacement or microevolutionary changes favoring susceptible strains. Future studies integrating whole-genome sequencing of archived isolates and antimicrobial consumption density metrics are needed to test this hypothesis and to determine whether the favorable trend can be sustained.

## Figures and Tables

**Table 1:** Demographic, clinical, and microbiological characteristics of patients with *S. aureus* clinical isolates, stratified by sex (June 2016–December 2025).

Variable	Total *n* (%)	Female *n* (%)	Male *n* (%)	*p*-Value
Number of isolates	6609 (100.0)	1692 (25.6)	4917 (74.4)	—
Department				
Nephrology	866 (13.1)	225 (13.3)	641 (13.0)	0.827
Orthopedics	564 (8.5)	91 (5.4)	473 (9.6)	<0.001
Internal Medicine	559 (8.5)	153 (9.0)	406 (8.3)	0.348
Neurosurgery	554 (8.4)	116 (6.9)	438 (8.9)	0.01
Adult Infectious Disease	413 (6.2)	102 (6.0)	311 (6.3)	0.699
Adult Intensive Care Unit (ICU)	376 (5.7)	87 (5.1)	289 (5.9)	0.282
General Surgery	298 (4.5)	69 (4.1)	229 (4.7)	0.352
Pediatric Intensive Care Unit (PICU)	211 (3.2)	75 (4.4)	136 (2.8)	0.001
Gynecology and Obstetrics	207 (3.1)	79 (4.7)	128 (2.6)	<0.001
Pediatric Emergency	191 (2.9)	58 (3.4)	133 (2.7)	0.15
Cardiac Surgery	185 (2.8)	32 (1.9)	153 (3.1)	0.011
Cardiology	179 (2.7)	60 (3.5)	119 (2.4)	0.018
Adult Emergency	171 (2.6)	50 (3.0)	121 (2.5)	0.313
Gastroenterology	160 (2.4)	33 (2.0)	127 (2.6)	0.169
HIV/AIDS Care Unit	160 (2.4)	20 (1.2)	140 (2.8)	<0.001
Others	1515 (22.9)	442 (26.1)	1073 (21.8)	—
Blood Culture	1330 (20.1)	330 (19.5)	1000 (20.3)	0.47
Bronchial Aspirate Culture	616 (9.3)	192 (11.3)	424 (8.6)	0.001
Surgical Wound Secretion Culture	579 (8.8)	128 (7.6)	451 (9.2)	0.048
Various Fluid Cultures not specified	486 (7.4)	146 (8.6)	340 (6.9)	0.024
Secretion Culture	469 (7.1)	75 (4.4)	394 (8.0)	<0.001
Tissue Culture	352 (5.3)	99 (5.9)	253 (5.1)	0.297
Abscess Culture	331 (5.0)	91 (5.4)	240 (4.9)	0.462
Tracheal Aspirate Culture	273 (4.1)	78 (4.6)	195 (4.0)	0.285
Sputum Culture	251 (3.8)	53 (3.1)	198 (4.0)	0.111
Peritoneal Fluid Culture	237 (3.6)	65 (3.8)	172 (3.5)	0.567
Urine Culture	230 (3.5)	53 (3.1)	177 (3.6)	0.404
Peripheral Blood Culture	192 (2.9)	40 (2.4)	152 (3.1)	0.145
Catheter Tip Culture	191 (2.9)	60 (3.5)	131 (2.7)	0.076
Wound secretion culture	188 (2.8)	50 (3.0)	138 (2.8)	0.821
Others	871 (13.2)	230 (13.6)	641 (13.0)	—
Clinical diagnosis				
Bloodstream infections	1873 (28.3)	464 (27.4)	1409 (28.7)	0.336
Surgical site infections	1248 (18.9)	257 (15.2)	991 (20.2)	<0.001
VAP	955 (14.4)	285 (16.8)	670 (13.6)	0.001
Not specified	493 (7.5)	149 (8.8)	344 (7.0)	0.017
Tissue not specified	351 (5.3)	98 (5.8)	253 (5.1)	0.342
Abscess site not specified	331 (5.0)	91 (5.4)	240 (4.9)	0.462
Hospital-acquired pneumonia	278 (4.2)	59 (3.5)	219 (4.5)	0.1
Dialysis-related peritonitis	237 (3.6)	65 (3.8)	172 (3.5)	0.567
Urinary tract infection	230 (3.5)	53 (3.1)	177 (3.6)	0.404
Community-acquired pneumonia	147 (2.2)	42 (2.5)	105 (2.1)	0.464
Septic arthritis	136 (2.1)	21 (1.2)	115 (2.3)	0.008
Meningitis	117 (1.8)	27 (1.6)	90 (1.8)	0.597
Pharyngitis	70 (1.1)	23 (1.4)	47 (1.0)	0.209
Pressure ulcer infection	53 (0.8)	23 (1.4)	30 (0.6)	0.005
Conjunctivitis	31 (0.5)	11 (0.7)	20 (0.4)	0.292
Others	59 (0.9)	24 (1.4)	35 (0.7)	—
Methicillin resistance				
MRSA	1395 (21.1)	279 (16.5)	1116 (22.7)	<0.001
MSSA	5214 (78.9)	1413 (83.5)	3801 (77.3)	

Note: Percentages for Total are calculated over *n* = 6609. Percentages for Female and Male columns are calculated within each sex group. *p*-values from Pearson’s chi-square test comparing proportions between sexes. VAP, ventilator-associated pneumonia; MRSA, methicillin-resistant *S. aureus*; MSSA, methicillin-susceptible *S. aureus*.

**Table 2:** Summary of trend analyses of MRSA (2016–2025).

Method	Parameter	Estimate	95% CI	*p*-Value
MRSA proportion (weekly)				
Mann–Kendall	*z*	−9.03	–	<0.001
	tau (Kendall)	−0.282	–	–
Theil-Sen	Median slope (per week)	−4.61 × 10^−4^	−5.60 × 10^−4^ to −3.64 × 10^−4^	<0.001
	Annual change	−2.4 pp/year	−2.9 to −1.9	–
Binomial GLM (logit)	OR per year	0.85	0.829 to 0.871	<0.001
	Marginal effect	−2.8 pp/year	–	–
MRSA incidence density (monthly, per 1000 PD)				
Mann–Kendall	*z*	−4.91	–	<0.001
	tau (Kendall)	−0.312	–	–
Theil-Sen	Annual change	−0.099/1000 PD/year	−0.135 to −0.063	<0.001
*S. aureus* incidence density (monthly)				
Mann–Kendall	*z*	−0.17	–	0.868

Note: pp, percentage points; PD, patient-days. *z* denotes the standardized Mann–Kendall test statistic. “–” indicates not applicable/not reported for that parameter (e.g., no CI or *p*-value). The binomial GLMs model MRSA counts as binomial outcomes with a logit link (see Section 4.4.1). The marginal effect approximates the annual change in MRSA proportion at the sample mean.

**Table 3:** MRSA and *S. aureus* incidence density per 1000 patient-days by COVID-19 phase, 2016–2025.

Phase	Months	Patient-Days	MRSA Cases	MRSA per 1000 PD	*S. aureus* per 1000 PD	Change vs. Pre-Pandemic
Pre-pandemic (2016–2020)	44	513,713	653	1.27	4.53	Reference
High circulation (2020–2022)	24	247,340	373	1.51	6.63	19%
Post-peak (2022–2025)	46	589,487	369	0.63	4.49	−50%
Overall	114	1,350,540	1395	1.03	4.89	—

Note: PD, patient-days. The binomial GLMs MRSA counts as binomial outcomes with a logit link (see Section 4.4.1). The marginal effect approximates the annual change in MRSA proportion at the sample mean. Corresponding incidence per 1000 admissions is provided in Supplementary Table S1.

## Data Availability

De-identified aggregated data supporting the findings of this study are available in Zenodo at https://doi.org/10.5281/zenodo.18382603.

## Supplementary Materials

The following supporting information can be downloaded at https://www.mdpi.com/article/10.3390/antibiotics15030242/s1, Table S1: *S. aureus* and MRSA incidence density per 1000 patient-days and per 1000 admissions by COVID-19 phase; Table S2: Sensitivity analysis: interrupted time series regression coefficients for monthly MRSA counts (114 observations) across three estimation methods; Figure S1: Weekly *S. aureus*, MRSA, and MSSA incidence per 1000 admissions with LOESS smoothing.

## Abbreviations

The following abbreviations are used in this manuscript:

AMR	Antimicrobial-Resistance
ATCC	American Type Culture Collection
BSIs	Bloodstream Infections
CI	Confidence Interval
CLSI	Clinical and Laboratory Standards Institute
COVID-19	Coronavirus Disease 2019
GAM	Generalized Additive Model
HAIs	Healthcare-Associated Infections
ICUs	Intensive Care Units
LOESS	Locally Estimated Scatterplot Smoothing
MIC	Minimum Inhibitory Concentration
MRSA	Methicillin-Resistant *Staphylococcus aureus*
MSSA	Methicillin-Susceptible *Staphylococcus aureus*
PACF	Partial Autocorrelation Function
PPE	Personal Protective Equipment
PICU	Pediatric Intensive Care Unit
STL	Seasonal-Trend Decomposition using Loess
SSIs	Surgical Site Infections
VAP	Ventilator-Associated Pneumonia
