# Supplementary material for: Trends, Seasonality, and the Impact of COVID-19 on Clinical Staphylococcus aureus and MRSA Isolates in Western Mexico (2016–2025): A Time-Series Analysis at a University Referral Hospital
Source: Antibiotics (Basel). 2026 Feb 25;15(3):242. doi: 10.3390/antibiotics15030242 (PMC13024594; doi:10.3390/antibiotics15030242)
Supplement: Supplementary file 1 [file antibiotics-15-00242-s001.zip › antibiotics-4147712-supplementary.pdf]

Supplementary Material

Supplementary Figures

Supplementary Figure S1. Weekly *Staphylococcus aureus* and MRSA incidence per 1,000 admissions with LOESS smoothing (span = 0.3), 2016--2025.

Supplementary Tables

Supplementary Table S1. Supplementary Table S1. *Staphylococcus aureus* and MRSA incidence density per 1,000 patient-days and per 1,000 admissions by COVID-19 phase, Hospital Civil "Fray Antonio Alcalde," 2016--2025.
Supplementary Table S2. Sensitivity analysis: interrupted time series regression coefficients for monthly MRSA counts (114 observations) across three estimation methods, Hospital Civil "Fray Antonio Alcalde," 2016–2025.

Supplementary Figure S1. Weekly *Staphylococcus aureus* and MRSA incidence per 1,000 admissions with LOESS smoothing (span = 0.3), 2016--2025. (a) Total S. aureus incidence per 1,000 admissions. (b) Weekly MRSA and MSSA incidence per 1,000 admissions. Dots represent observed weekly values; solid lines indicate LOESS-smoothed trends with 95% confidence intervals (shaded ribbons). Dashed vertical lines indicate COVID-19 onset (March 2020, orange) and post-Omicron transition (March 2022, green)

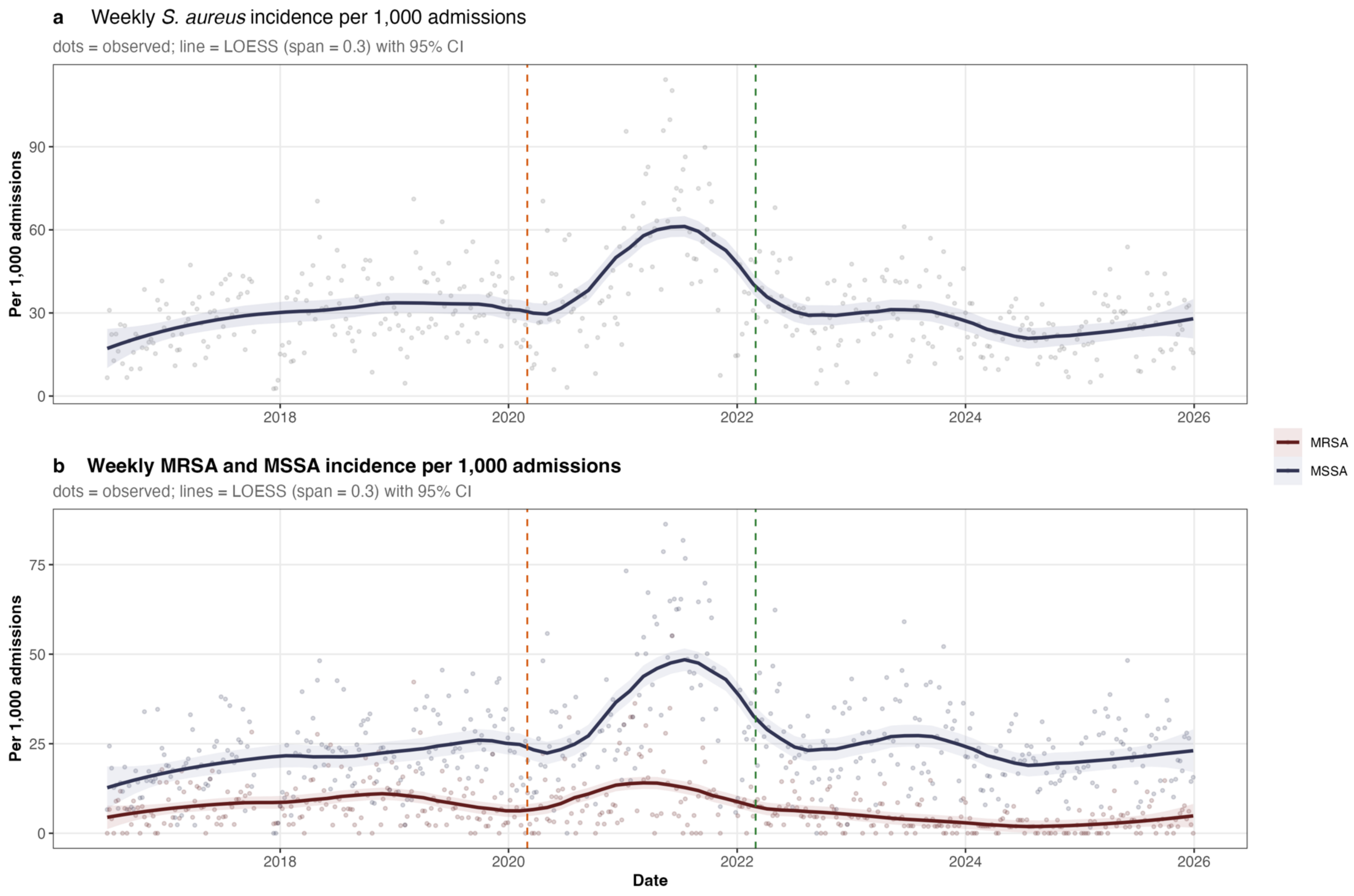

Supplementary Table S1. *Staphylococcus aureus* and MRSA incidence density per 1,000 patient-days and per 1,000 admissions by COVID-19 phase, Hospital Civil "Fray Antonio Alcalde," 2016--2025.

phase	Months	Admissions	Patient days	SA cases	MRSA cases	SA per 1000 PD	SA per 1000 Adm	MRSA per 1000 PD	MRSA per 1000 Adm
Pre-pandemic	44	82245	513713	2325	653	4.53	28.27	1.27	7.94
High circulation	24	38027	247340	1640	373	6.63	43.13	1.51	9.81
Post-peak	46	98031	589487	2644	369	4.49	26.97	0.63	3.76
Overall	114	218303	1350540	6609	1395	4.89	30.27	1.03	6.39

Supplementary Table S2. Sensitivity analysis: interrupted time series regression coefficients for monthly MRSA counts (114 observations) across three estimation methods, Hospital Civil "Fray Antonio Alcalde," 2016–2025.

**Coefficient**	**OLS Est. (SE)**	**OLS p**	**GLS-AR(1) Est. (SE)**	**GLS-AR(1) p**	**NW Est. (SE)**	**NW p**
β0 Intercept	11.811 (2.368)	< 0.001	11.669 (2.757)	< 0.001	11.811 (2.327)	< 0.001
β1 Pre-pandemic slope	0.135 (0.092)	0.145	0.137 (0.106)	0.2	0.135 (0.106)	0.207
β2 High-circ level shift	−3.624 (3.978)	0.364	−3.184 (4.536)	0.484	−3.624 (4.440)	0.416
β3 High-circ slope change	−0.020 (0.245)	0.934	−0.059 (0.284)	0.837	−0.020 (0.354)	0.954
β4 Post-peak level shift	−6.103 (3.834)	0.114	−5.506 (4.375)	0.211	−6.103 (5.760)	0.292
β5 Post-peak slope change	−0.231 (0.243)	0.345	−0.199 (0.281)	0.481	−0.231 (0.323)	0.477
AR(1) ϕ	—	—	0.161	—	—	—
Durbin–Watson	1.661	0.022	—	—	—	—
Joint test (H0: β3 + β5 = 0)	F = 4.00	0.048	—	—	—	—

Monthly MRSA counts (114 observations) were analyzed as a sensitivity check for the weekly primary analysis (Table 4, 476 observations). The model specification was identical: $Y = \beta_0 + \beta_1 \cdot \text{time} + \beta_2 \cdot \text{high\_circ} + \beta_3 \cdot \text{t\_high} + \beta_4 \cdot \text{post\_peak} + \beta_5 \cdot \text{t\_post}$. Phase boundaries were defined as high viral circulation from March 2020 and post-peak stabilization from March 2022. OLS, ordinary least squares; GLS-AR(1), generalized least squares with first-order autoregressive correlation; NW, Newey–West heteroscedasticity- and autocorrelation-consistent standard errors. The joint test evaluates $H_0: \beta_3 + \beta_5 = 0$(OLS model)
